# Evaluating measures to assess loneliness in autistic adults

**DOI:** 10.1177/13623613231217056

**Published:** 2023-12-25

**Authors:** Kana Grace, Anna Remington, Jade Davies, Laura Crane

**Affiliations:** University College London, UK

**Keywords:** adults, autism, loneliness, measurement

## Abstract

Several measures have been developed to assess loneliness in the general population. Here, we examined if, and how accurately, widely-used measures capture the experience of loneliness in autistic adults. A total of 203 autistic participants from the UK completed an online survey, which included two widely-used loneliness questionnaires: the University of California, Los Angeles (UCLA) Loneliness Scale Version 3 and the Social and Emotional Loneliness Scale for Adults (SELSA). Participants completed the measures and provided their views on how well they captured their experiences of loneliness. A direct measure, asking participants how often they felt lonely, was also completed. Results demonstrated that scores on the UCLA and SELSA questionnaires positively correlated with the direct measure of loneliness, suggesting that these tools accurately index autistic experiences of loneliness. However, qualitative responses suggested that participants found both scales difficult to complete. Using reflexive thematic analysis, we identified three themes that encapsulated participants’ views: (1) failing to distinguish between the characteristics/experiences of loneliness and being autistic, (2) loneliness is not a static trait and (3) inappropriate wording. Given that our sample of autistic participants tended to prefer the UCLA scale to the SELSA, we provide initial recommendations around how the UCLA scale could be adapted for autistic adults.

**Lay abstract:**

There has been increasing interest in research on loneliness in autistic adults. Much of this research has involved giving autistic adults widely-used questionnaires that are thought to measure how lonely people are. However, these questionnaires have been developed for the general public. We do not know whether these questionnaires accurately measure how lonely autistic adults are. We asked 203 autistic adults to complete an online survey that included two widely-used loneliness questionnaires: (1) the University of California, Los Angeles (UCLA) Loneliness Scale Version 3 and (2) the Social and Emotional Loneliness Scale for Adults (SELSA). We also asked participants to rate how lonely they were from 1 (often/always) to 5 (never). Participants were then asked to give their views on the questionnaires (e.g. what they thought was good, and what they thought was not so good about them). We found that the scores on the UCLA scale and the SELSA aligned with participants’ ratings of how lonely they were, which suggests that these two questionnaires accurately measure loneliness in autistic people. However, our participants also identified several ways to improve the questionnaires. This included (1) better distinguishing the characteristics/experiences of loneliness from those of being autistic; (2) better reflecting how loneliness may change at different times and in different contexts and (3) making the phrasing of the questions clearer. Overall, our autistic participants tended to prefer the UCLA scale to the SELSA. Therefore, we present some recommendations about how the UCLA scale could be changed to be more suitable for autistic people.

## Introduction

Loneliness is a distressing emotional state (Perlman & Peplau, 1981; Wang et al., 2017), associated with a range of negative physical and mental health outcomes (Cacioppo et al., 2015; Wang et al., 2018). One group that may be particularly at risk of experiencing loneliness is the autistic population (Grace et al., 2022). Historical stereotypes incorrectly portrayed autistic people as being intentionally isolated and disinterested in social connection (Asperger, 1944; Chevallier et al., 2012; Kanner, 1943). However, autistic people vary considerably in the extent to which they desire social interaction. Some may prefer to spend most of their time with animals (e.g. Holliday-Willey, 2014) or engaging with interests and passions (e.g. Grove et al., 2018), while others strive for social interactions with other people despite potentially experiencing challenges in forming and maintaining relationships with others (Sedgewick & Pellicano, 2019). These challenges can lead to reduced satisfaction in one’s interpersonal relationships (e.g. lower friendship quality and/or feeling that one is on the periphery of social networks) and result in increased loneliness for autistic people (Sumiya et al., 2018; White & Roberson-Nay, 2009; Whitehouse et al., 2009).

Unlike phenomena such as social isolation, which can be more objectively quantified, measuring loneliness is challenging due to its subjective nature (de Jong Gierveld & Havens, 2004; Wang et al., 2017). Several standardised measures have been developed to identify the extent of loneliness that a person experiences and whether it changes over time. These measures encompass various aspects of loneliness (e.g. intimate, relational or collective loneliness) and employ both indirect (e.g. asking about emotions associated with loneliness) and direct (e.g. asking explicitly about feelings of loneliness) scales. Within previous literature on autistic loneliness, commonly-used measures include the University of California, Los Angeles (UCLA) Loneliness Scales and the Social and Emotional Loneliness Scale for Adults (SELSA; DiTommaso & Spinner, 1993). Using these existing measures, autistic adults tend to report higher levels of loneliness than non-autistic adults. For example, Russell (2020) found that autistic adults scored on average 10.8 points higher than non-autistic adults on the UCLA Loneliness Scale Version 3 (Russell, 1996), and mean scores on all subscales on SELSA (Schiltz et al., 2021) were higher for autistic adults than general population norms (e.g. 56 vs 43 for romantic, 25 vs 21 for family and 47 vs 31 for social subscales, DiTommaso & Spinner, 1993) (see Grace et al., 2022, for a review). Both these measures were, however, created and validated for use with the general population.

Using existing measures to assess autistic experiences of loneliness might result in inaccurate findings, as they may not reflect the unique way autistic people experience loneliness (Jones, 2022; Nicolaidis et al., 2020; Stacey & Cage, 2023). For example, some frequently-used loneliness measures ask about friendships, but autistic people may understand friendship differently from non-autistic people (see Sedgewick & Pellicano, 2019). Furthermore, autistic people may interpret scale items differently from the general population, for whom the existing measures were intended (Mason et al., 2022; McConachie et al., 2018). Specific examples of this are discussed later in this introduction. Finally, autistic adults may experience challenges with decision-making in responding to questionnaire measures. Indeed, Stacey and Cage (2023) caution that researchers need to ensure that questionnaire measures are accessible for autistic people, to ensure meaningful participation in research.

The importance of creating bespoke versions of screening measures can be seen in research on psychological attributes akin to loneliness. One such example is quality of life, where the most frequently-used measure, the brief version of the World Health Organization Quality of Life scale (WHOQoL-BREF, Skevington et al., 2004), was adapted for use with autistic adults (Mason et al., 2022; McConachie et al., 2018, 2020). The development of the autism-specific ASQoL involved consultation with autistic adults, qualitative investigation of autistic adults’ views on existing items and validation of new items with a large group of autistic people (McConachie et al., 2018, 2020). Highlighting the need for a bespoke measure, the researchers found that autistic adults interpreted several items from the original WHOQoL-BREF (e.g. social aspects, bodily appearance, mental health) differently to people in the general population.

Similarly, tailored tools have been developed to assess suicidality in autistic people. Using interviews and questionnaires, Cassidy et al. (2020) established that certain items on the Suicidal Behaviours Questionnaire-Revised (SBQ-R; Osman et al., 2001) were interpreted differently by autistic compared to non-autistic adults: specifically concerning likelihood of a future suicide attempt and communication of threat of suicide attempt to others. These findings were used to create and validate the Suicidal Behaviours Questionnaire – Autism Spectrum Conditions (SBQ-ASC; Cassidy et al., 2021). Likewise, Rodgers and colleagues have created autism-specific anxiety measures for autistic children (through consulting with their parents; Rodgers et al., 2016) and for autistic adults (through consulting with autistic adults and professionals; Rodgers et al., 2020). Taken together, the work done in these three areas highlights the value of including the input of autistic people to create measures that are specifically tailored to the autistic population and offers best-practice recommendations for the adaptation process (Nicolaidis et al., 2020).

To our knowledge, only one study has used an autism-specific measure to assess loneliness. Merkler (2007) modified the Peer Network and Dyadic Loneliness Scale (PNDLS) (Hoza et al., 2000) to create an *Isolation and Affect Measure* assessing social network and dyadic loneliness in autistic adults. This measure included 28 items; 15 on social network isolation (e.g. I fit in with a group of people) and 13 on dyadic isolation (e.g. I have a best friend). Each item was answered using a five-point scale from 1 (not at all) to 5 (absolutely). Respondents were also asked to select an emotional response to each item (happiness, sadness, anger, anxiety, loneliness) and rate the intensity of the emotion using a five-point scale (1 = not at all to 5 = most intense). The measure generates scores on four subscales (dyadic isolation, dyadic distress, social network isolation and social network distress), and its validity was demonstrated via confirmatory factor analysis and correlation between the Isolation and Affect measure and other similar measures (e.g. SELSA). Merkler (2007) did not, however, involve autistic adults in developing this loneliness measure, and the measure has not been used beyond their study. Furthermore, Merkler (2007) did not establish whether there was a need for an autism-specific measure of loneliness, or whether existing tools adequately captured the autistic experience.

The current study takes the first steps towards determining whether there is a need for an autistic-informed, bespoke measure to assess autistic people’s experiences of loneliness. Specifically, we examine (1) if, and how accurately, existing loneliness measures capture the experiences of loneliness in autistic adults and (2) autistic adults’ views/experiences of two widely-used loneliness measures. Based on previous studies on quality of life, suicidality and anxiety among autistic people, we expected that existing loneliness measures might not accurately capture the experiences of loneliness for autistic adults and that autistic adults might have difficulties completing existing questionnaires.

## Methods

### Community involvement statement

This study was led by an autistic researcher (who was involved in every stage of the research process, from study conception to dissemination), in collaboration with non-autistic colleagues. All authors view autism from a social (as opposed to a medical) model of disability and align with the neurodiversity paradigm. Six autistic adults also provided feedback to the research team on an early version of the online survey used in the study.

### Participants

Autistic adults (diagnosed or self-identified), older than 18 years and currently living in the UK were recruited via organisations and social groups for autistic adults, social media and our research centre database. A total of 294 autistic adults engaged with the survey, but seven were excluded: Five were not autistic, and two did not live in the UK. Only those who completed the survey in full were included (i.e. those who partially completed the survey were excluded, n = 84). The final sample comprised 203 respondents. Most were formally diagnosed (predominantly in adulthood), female and from a White ethnic background. Ages ranged from 18 to 73 years, and many participants had co-occurring conditions, most commonly mental health concerns (see Table 1 and Supplemental Material).

**Table 1.** Participant demographics (n = 203).

Demographic variables	n (%)
Autism diagnosis	
Formally diagnosed	172 (84.7)
Self-identified and in process of obtaining a diagnosis	14 (6.9)
Self-identified but not seeking a diagnosis	17 (8.4)
Age	
M: years (SD)	40.7 (12.5)
Aged 24 and younger	28 (13.8)
Aged 25–34	35 (17.2)
Aged 35–44	58 (28.6)
Aged 45–54	50 (24.6)
Aged 55–65	28 (13.8)
Aged 66 and above	4 (2.0)
Gender	
Male (including transgender male)	65 (32.0)
Female (including transgender female)	116 (57.1)
Non-binary	17 (8.4)
Other/prefer not to say	5 (2.5)
Ethnicity	
White (including British, Irish or any other White background)	183 (90.1)
Black or Black British Caribbean (including the Caribbean, African or any other Black background)	1 (0.5)
Asian or Asian British (including Indian, Pakistan, Bangladesh or any other Asian Background)	4 (2.0)
Mixed (e.g. White and Asian; or any other mixed background)	10 (4.9)
Other/prefer not to say	(2.5)

### Materials

#### Survey

Participants completed a bespoke survey, presented online via Qualtrics (https://www.qualtrics.com/). The survey was developed by K.G. (an autistic autism researcher) with advice from L.C. Six autistic adults, not involved with the design of the survey, provided feedback and suggestions on the first iteration of the survey. In response, one question (on changes in experiences of loneliness over time) was added to the survey. The final survey comprised three sections. First, participants were asked to provide demographic information (e.g. age, gender, diagnostic status, education experience). Second, participants were asked to complete two measures of loneliness (identified as widely used in a previous systematic review; Grace et al., 2022): the UCLA Loneliness Scale Version 3 (Russell, 1996) and SELSA (DiTommaso & Spinner, 1993). The order of the measures was counterbalanced across participants. After completing each measure, participants were asked to provide comments in open-text boxes to respond to the following question: ‘if you would like to, please tell us what you think about this loneliness questionnaire (e.g. what is good about it, what is not-so-good about it, and any improvements that could be made to better reflect your experiences of loneliness)’. Finally, participants were asked to complete a direct measure of loneliness (Office for National Statistics, 2018); to enable us to assess how accurately the UCLA scale and SELSA measured loneliness in autistic adults.

### Measures

#### UCLA Loneliness Scale Version 3

This self-report, unidimensional scale assesses the frequency and intensity of experiences of loneliness (Russell, 1996). The scale does not specify a time frame for respondents when reflecting on their experience of loneliness (Cramer & Barry, 1999). The scale comprises 20 items with four response options (1 = never, 2 = rarely, 3 = sometimes, 4 = always) (e.g. ‘how often do you feel alone’, ‘how often do you feel left out’). Scores are summed to create a total, ranging from 20 to 80, with higher scores indicating higher loneliness. The measure has good internal consistency (Cronbach’s alpha = 0.89–0.94) and test-retest reliability (*r* = 0.73) in the general population (Russell, 1996). Grace et al. (2022) reported that this scale was previously used in five studies of autistic adults (Brooks, 2014; Hedley et al., 2018; Hillier et al., 2018; Jantz, 2011; Russell, 2020); however, internal consistency was not reported in any of these studies. In the current study, the measure had good internal consistency (Cronbach’s alpha = 0.90).

#### Social Emotional Loneliness Scale for Adults (SELSA)

This self-report, multidimensional scale assesses the frequency and intensity of experiences of intimate and relational aspects of loneliness (DiTommaso & Spinner, 1993). The article originally reporting on the SELSA scale (DiTommaso & Spinner, 1993) mentions three options to present to participants when reflecting on their experiences of loneliness (i.e. the last 2 years, the last 2 weeks, the next 2 years). However, to maintain consistency with the UCLA scale, we did not include a specific time frame for our respondents. The scale includes 37 items, each answered on a seven-point scale (1 = strongly disagree to 7 = strongly agree) (e.g. ‘I don’t have a friend(s) who understands me, but I wish I did’, ‘I have a lot in common with others’). The scale comprises three subscales (romantic, family and social) with scores ranging from 12 to 84 for the romantic subscale, 11 to 77 for the family subscale and 14 to 98 for the social subscale. Total scores range from 37 to 259, with higher scores indicating higher loneliness. The three subscales of the measure have good internal consistency in the general population (Cronbach’s alpha = 0.89–0.93) (DiTommaso & Spinner, 1993). Grace et al. (2022) reported that this scale was previously used in five studies of autistic adults (Bourdeau, 2020; Gantman et al., 2012; McVey et al., 2016; Merkler, 2007; Schiltz et al., 2021), but only McVey et al. (2016) reported on the internal consistency of the measure (Cronbach’s alpha = 0.71). In the current study, the three subscales of the measure had good internal consistency (Cronbach’s alpha = 0.92 for the social subscale, 0.93 for the family subscale and 0.94 for the romantic subscale).

#### Direct measure of loneliness

This self-report unidimensional scale asks respondents ‘(how often) do you feel lonely?’ and offers five answer choices (1 = often/always, 2 = some of the time, 3 = occasionally, 4 = hardly ever, 5 = never) (Office for National Statistics, 2018). The UK government recommends using the direct measure in the general adult population as a national indicator of loneliness (Office for National Statistics, 2018), in addition to indirect measures.

### Procedure

Ethical approval was attained via the Department of Psychology and Human Development at IOE, UCL’s Faculty of Education and Society. Before beginning the online survey, we presented participants with information about the research, including a link to view the whole survey in advance. All participants gave their written consent on an online form. Given the sensitivity of the topic of loneliness, participants were given contact details of who they could get in touch with if the study raised any negative feelings (e.g. Samaritans, who have a text and phone service), and this appeared on every page of the survey. Data were collected between November 2019 and January 2020 (approximately 6 weeks). Please note that the work presented was part of a broader study also assessing definitions and experiences of loneliness, but these data are not presented here (see Umagami, 2023, for details).

### Analyses

#### Quantitative analyses

To determine whether there were differences in loneliness scores between formally-diagnosed and self-identifying autistic adults (given the debates around whether adults who self-identify as autistic should be included in autism research; see Overton et al., 2023, for an overview), we ran independent samples t-tests (or non-parametric equivalents). To answer our research questions, we ran correlations to determine whether scores on each of the three loneliness measures were related to one another.

#### Qualitative analyses

We used reflexive thematic analysis (Braun & Clarke, 2006, 2019; Clarke & Braun, 2013) and took an inductive approach to analyse autistic adults’ views on the UCLA and SELSA scales. Coding and theme development proceeded in a recursive manner, led by K.G. (an autistic researcher) together with J.D. (a non-autistic researcher) and with advice from L.C. (another non-autistic researcher). We followed recommendations of Nowell et al. (2017) to ensure trustworthiness of the data, including: prolonged engagement with the data, documentation of team discussions regarding codes and themes, repeated diagramming to establish theme connections, ongoing team-wide reviews of themes and reviewing report drafts to reach consensus. To ensure that the analysis acknowledged differing perspectives from the majority voice, we also conducted a negative case analysis (Hanson, 2017): purposefully looking for data that contradicted the themes. However, instances of participant responses that contradicted the final themes were rare.

## Results

### Quantitative results

#### Loneliness scores for formally-diagnosed and self-identifying autistic adults

There were no differences in loneliness scores on any measure between formally-diagnosed and self-identifying autistic adults (*p* > 0.24; see Supplemental Material for full outputs). We therefore conducted the subsequent analyses on the entire participant group, not distinguishing between groups on the basis of diagnostic status.

#### Overall loneliness scores

Participants’ scores on the UCLA, SELSA and direct measure of loneliness are presented in Table 2. In the absence of a comparison group within our study, we reflect on how these data compare to scores in the general population in the Discussion section.

**Table 2.** Loneliness scores from the UCLA, SELSA and direct measure.

Measure	M (SD)	Range	N (%)
UCLA scale			
Total score (min 20, max 80)	60.1 (10.8)	26–80	
SELSA			
Total score (min 37, max 259)	148.9 (43.6)	51–247	
Romantic subscale (min 12, max 84)	47.9 (22.2)	12–84	
Family subscale (min 11, max 77)	37.1 (16.8)	11–77	
Social subscale (min 14, max 98)	63.9 (19.0)	23–98	
Direct measure			
Total score (min 1, max 5)	2.2 (1.1)	1–5	
Often/always			72 (35.5)
Some of the time			61 (30.1)
Occasionally			40 (19.7)
Hardly ever			22 (10.8)
Never			8 (3.9)

UCLA: University of California, Los Angeles; SELSA: Social and Emotional Loneliness Scale for Adults.

#### Correlations between the loneliness measures

The three loneliness measures were positively correlated with one other. As seen in Table 3, these data suggest that all measures index the same construct and that the UCLA and SELSA scales align with autistic adults’ subjective experiences of loneliness.

**Table 3.** Correlations among the three self-report loneliness measures.

	SELSA	Direct measure
UCLA	*r* = 0.79 *p* < 0.001	*r* = –0.53 *p* < 0.001
SELSA	—	*r* = –0.50 *p* < 0.001

UCLA: University of California, Los Angeles; SELSA: Social and Emotional Loneliness Scale for Adults.

### Qualitative results

#### Autistic adults’ views on the loneliness measures

**Figure 1.**
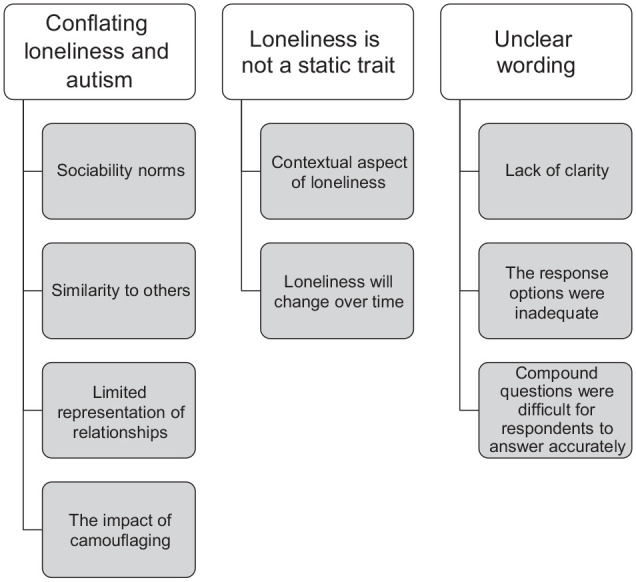
Thematic map of autistic adults’ views on the loneliness measures.

Overall, respondents noted the uncomfortable nature of loneliness questionnaires (‘It might be upsetting to answer for someone who doesn’t have a partner or a good relationship with their family’, participant 18, henceforth P18). Some participants (n = 32) spontaneously expressed a preference for one scale over the other, and these participants tended to prefer the UCLA scale (n = 28) over the SELSA (n = 4). Respondents generally appreciated the simple and relatable nature of the UCLA scale: ‘This questionnaire is excellent and is far better than the previous questionnaire that is on this survey. You can relate to the questions and answer them easier’ (P43). There was, however, a clear view that adjustments were needed to make either questionnaire fully suitable for use with the autistic population. Taking comments across both loneliness measures, three themes were identified (see Figure 1, as well as Supplemental Material, for a list of themes and subthemes, with example quotes). Quotes are presented verbatim, including any spelling/typographic errors.

#### Theme 1: conflating loneliness and autism

Participants felt that some items on both scales could lead to incorrect assumptions being made about one’s experience of loneliness, since participants felt that there were underlying assumptions regarding aspects of human behaviour and how these contribute to loneliness; aspects that are perhaps different for autistic people.

These assumptions centred around four aspects. First, several questions were based on *sociability norms* (e.g. how often do you feel outgoing and friendly? UCLA Q9). Participants expressed that response options should be able to capture that one may not desire lots of friends/partners or want to engage in such relationships. In addition, participants noted an incorrect presumption that being alone was a negative experience that contributes to loneliness: ‘[The UCLA scale] assumes that everyone wants to be sociable and that they derive pleasure from the company of others. Some of us don’t. Or don’t all the time’ (P100). Participants explained how such assumptions could lead to inaccurate results: ‘where [the UCLA scale] says “How often do you feel isolated from others?”, I’ve said “Often”, but for me that’s the goal. Managing to avoid other people is success to me, but I think someone reading this survey would possibly read my answer as a negative instead of the positive I see it as’ (P46). On the SELSA, participants explained how many questions assumed that the respondent wanted to have friends/groups of friends/romantic partners and that there was the underlying assumption that somebody who does not have these things is therefore lonely (e.g. I’m not part of a group of friends and I wish I were. SELSA Q37). A participant explained: ‘they referred to a group of friends and although I have several friends they are not a group together but I am happy with this’ (P62).

Second, participants highlighted that various items asked about *similarity to others* (e.g. how often do you feel that you have a lot in common with the people around you? UCLA Q6; I feel ‘in tune’ with others. SELSA Q28) and used responses as indicators of social connectedness. As a minority neurotype, autistic people felt that they may be less similar to those around them but that this was not necessarily a precursor to loneliness: ‘I never have a lot in common with people around me because they aren’t ND [neurodivergent] and don’t share my way of looking at the world. That doesn’t mean that I’m lonely, it’s just a statement of fact!’ (P61). Another participant detailed: ‘Answering “often” to [how often do you feel that no one really knows you well? UCLA Q13] might look like I’m lonely, but I’ve never felt like anyone understands me and I have no expectation that anyone will’ (P66). Regarding the SELSA, a participant explained that ‘Not feeling “in tune” with others is more of an autistic thing than a loneliness thing for me’ (P125).

Third, participants noted that the questionnaires presented a *limited representation of relationships*, reflecting an assumption that loneliness is associated with certain types of social relationships and overlooking how the nature of relationships might differ for autistic people. For example, they highlighted that the questionnaire did not consider relationships that were not in close physical proximity (i.e. online friendships) but may still play an important role in reducing loneliness: ‘it’s hard to answer (the UCLA scale) because my best friends are online friends’ (P89), ‘I feel that this section (in the SELSA) doesn’t account for long distance friendships’ (P105). In addition to online relationships, autistic adults mentioned that the questionnaires overlooked non-human relationships: ‘I also am less lonely because I’m around my pets, and that isn’t considered here (UCLA scale)’ (P23), ‘Non-humans (pets) can provide companionship and thus reduce loneliness (but the SELSA does not consider this)’ (P205). Some participants felt that the questionnaires missed the role of professional connections: ‘Some of my contact with supportive others is from professionals and not informal social networks (but the SELSA does not consider this)’ (P85).

Finally, respondents highlighted how items on both scales did not consider *the impact of camouflaging*: that autistic adults may mask their true preferences and preferred behaviours as a way to ‘fit in’ with the neurotypical population, thereby skewing some of the results: ‘. . . I am able to do a lot of those things [described on the UCLA scale], however I don’t enjoy them, I don’t want to do them but feel as though I have to so that I am considered “normal” Its draining’ (P40). Similarly, participants explained how difficulties in distinguishing between their real feelings and masking made it difficult for them to answer questions on the SELSA: ‘I also struggled with some of these questions because it’s hard to unpick how I feel and how I perform “feeling”. I’d struggle to say I don’t feel part of my family because being part of my family [is] how society expects me to feel’ (P140).

#### Theme 2: loneliness is not a static trait

Participants explained that loneliness can change over time, and depending on who they are with, but that the questionnaires did not account for this more *contextual aspect of loneliness*: ‘A lot depends on the mood or situation of others at the time when I’d like their support’ (P63, reflecting on the UCLA scale). Similarly, participants felt that the questions in the UCLA scale did not consider the distinct experiences of professional and personal settings: ‘At work (both my paid work and my voluntary work), I tend to feel supported by those around with and we have a common interest but they are not my friends’ (P36). Participants also explained how *loneliness will change over tim*e: ‘feelings can vary according to life experiences, and day to day ups and downs’ (P135, reflecting on the SELSA), ‘my feelings seem to vary so much depending on how tired/anxious/depressed I am feeling’ (P186, reflecting on the UCLA scale).

#### Theme 3: unclear wording

The final theme centred on issues with the format of various questions. While one participant felt that the UCLA scale was ‘worded well’ (P45), many noted a *lack of clarity* in some of the items (e.g. regarding undefined terms such as ‘people’, ‘friends’ and ‘family’) (e.g. ‘the reference to “people” (in the UCLA scale) is a bit too general’, P169). Although some participants liked how both questionnaires did not explicitly refer to specific relationships, many felt that this led to a loss of nuance regarding individual relationships: ‘When (the SELSA) talks about family, for me that’s my mother. As an adult man this probably isn’t what the questionnaire means to ask about. I wish I had a family – a wife and kids, but this isn’t captured by the questionnaire’ (P60). Instead, participants felt that questions should target specific members (e.g. give a clear explanation who family is referring to) or give the option for free text: ‘all questions (in the SELSA) should be worded absolutely as clearly as possible so there is as little room for multiple interpretations of the question as possible and answer options should also be as clear as possible’ (P171).

Respondents highlighted that asking about all relationships together may skew the results of the survey (i.e. they may score very highly as they have one particularly good relationship but still feel lonely as they lack other relationships): ‘I have an amazing husband who supports me in all of the above which is why I’ve put sometimes. Outside of him I would have answered most of the questions more negatively’ (P98). Participants noted that non-literal language/idioms were also unhelpful. For example, regarding the item ‘I feel “in tune” with others’ (SELSA, item 28) a participant explained: ‘First, some autistic people might have issues with “in tune” – this isn’t a questionnaire about group music-making’ (P91).

Second, there were concerns that *the response options were inadequate*. For example, one participant detailed: [in the UCLA scale] ‘What exactly is the difference between often and rarely?^1^ Does never really mean never? Or is it a fuzzy never?’ (P60). Furthermore, participants felt that there were situations where their real experiences could not be explained using the four terms that were provided. Respondents suggested using a 10-point scale or even just providing clearer definitions (i.e. sometimes = happens 50% of the time). Participants noted that for some items, there needed to be a ‘not applicable’ option: ‘some of the questions [in the SELSA] were not applicable to me as I do not have a partner. A n/a option might be helpful’ (P178).

Third, particularly in the SELSA, *compound questions were difficult for respondents to answer accurately*. This issue was particularly salient with questions concerning romantic partners, whereby those without a romantic partner were unsure how to appropriately answer the question as their answer could be perceived in two ways: either that they were dissatisfied with their relationship or that they did not have a romantic partner. Issues were also raised about compound questions regarding friendships: ‘These are two separate statements that I felt I’d like to comment on separately: (1) not having friends I felt close to (2) wishing I did. Though I don’t think it affected my scoring, I didn’t like having to give a single answer to two statements at once’ (P140). Although many autistic adults emphasised unclear wording in the questionnaires, it should be noted that one participant felt that the UCLA scale was ‘worded well’ (P45).

## Discussion

In the present study, we offer the first direct investigation into the appropriateness of existing loneliness measures for autistic adults. To do so, we asked participants to complete and give their views on measures of loneliness that are frequently used with the general population. The autistic adults in our study scored higher on all measures of loneliness than general population samples in existing literature. For example, on the UCLA scale, our participants had a mean score of 60, compared to 39 reported for the general population (Brooks, 2014). Similarly, on the SELSA, all subscales were higher for participants in the present study than the general population, particularly the social subscale (48 vs 43 for romantic, 37 vs 21 for family and 64 vs 31 for social subscales, DiTommaso & Spinner, 1993). Furthermore, on the direct measure of loneliness, most of our participants reported feeling lonely often/always or some of the time (66%) compared to 21% of the general population (U.K. Department for Digital Culture Media & Sport, 2018). These results align with prior studies demonstrating higher levels of loneliness in autistic, compared to non-autistic, people (Grace et al., 2022; Hymas et al., 2022). We should be cautious, however, at drawing conclusions given that the general population groups cited earlier may not be matched to our participants on key demographics.

As with many autistic adult samples recruited online (see Rødgaard et al., 2022; Rubenstein & Furnier, 2020), participants in this study were not representative of the entire autistic population. For example, our participants were largely employed or in education and had gained higher educational qualifications. Such characteristics might suggest that our participants have fewer needs and are not likely candidates for support, yet our participants still experienced higher levels of loneliness. Autistic people could face various challenges in adulthood as a result of being perceived as having fewer needs (Kapp, 2018). For example, in a study examining mental health, autistic young people expressed how they felt ‘too normal to be seen as different, but not normal enough to fit in’, which meant they were overlooked for support, and this led to mental health difficulties (Crane et al., 2019). A similar paradox has been noted regarding social experiences, where autistic children with *higher* levels of social skills are more likely to be victims of bullying (Sedgewick & Pellicano, 2019). The authors note that this may be because peers have higher expectations for such children and then punish them when these expectations are not met. This pattern of experiences could likely apply to loneliness.

To ascertain how lonely autistic people are, it is essential that we can be confident in the measurement tools used to assess loneliness. Both the UCLA scale and SELSA correlated with the direct measure of loneliness, suggesting that the measures aligned with autistic adults’ subjective experiences of loneliness. As such, both measures may be sufficient to provide a marker of loneliness for autistic people. Yet qualitative data highlighted several ways in which autistic adults experienced difficulties when completing the measures and felt that their scores might not represent the nuances of their actual experiences of loneliness. These issues might lead to an over- or under-estimation of loneliness in absolute terms, even if the scores correlated with other measures.

First, many concerns raised by our participants centred around how the measures did not take into account autistic experiences but were based on assumptions about non-autistic norms. For example, the measures asked about similarity to others and frequency of being alone – both of which might be different for autistic people compared to non-autistic people, without necessarily being a marker of loneliness. The issue of measures not reflecting autistic experiences has led to participants feeling misunderstood about their experiences of loneliness and has also been highlighted in studies on measures beyond the field of loneliness research. In their review of studies that adapted survey instruments for autistic adults, Nicolaidis et al. (2020) reported that existing measures did not capture autism-specific aspects of the constructs (e.g. not considering sensory barriers in accessing health care). Similarly, regarding quality of life and suicidality research, autistic adults were reported to interpret some questionnaire items differently from the general population (Cassidy et al., 2020; Mason et al., 2022). With respect to the latter, the item asking whether respondents have communicated their suicidal thoughts to others was considered irrelevant in terms of their suicidality by autistic adults (Cassidy et al., 2020).

Regarding the present study, autistic adults described that camouflaging (see Cook et al., 2021) was not considered in widely-used loneliness scales. This omission could mean that autistic adults score differently on the measures depending on whether they respond to the items thinking about when being true to themselves or when camouflaging to meet others’ social expectations. For example, in relation to item 9 on the UCLA scale (how often do you feel outgoing and friendly?), autistic adults may respond with often/always when thinking about their behaviour when camouflaging but respond with rarely/never when thinking about their behaviour when not camouflaging. Relying on such measures could also overlook the underlying social experiences of autistic adults: that social interaction can be exhausting (Elmose, 2020). Not considering camouflaging appears to be prevalent in other questionnaires that attempt to link behaviours with intentions. For example, in a recent study of autistic adults’ decision-making with regards to research questionnaires, Stacey and Cage (2023) touched upon the impact of ‘social influence’, highlighting how the autistic adults in their sample felt that questionnaires often did not consider camouflaging (e.g. in hiding how they feel to enable them to carry out day-to-day tasks). Such autism-specific experiences are thus essential to consider in questionnaire development.

Second, autistic adults in this study reported several ways in which items on the UCLA scale and SELSA were not clearly worded, including inadequate response options or compound questions that were challenging to parse, the use of idioms and inability to account for varying loneliness over time and across contexts. These issues led to autistic adults fearing that their experiences of loneliness would be misunderstood. As with concerns about the uniqueness of the autistic experience, this issue is not exclusive to loneliness measures. Studies of quality of life, suicidality and the use of measures with autistic populations in general have reported difficulties with questionnaire wording. As with our findings, these concerns included challenging terms/phrases/sentence structure (e.g. the term, ‘get around’), unclear/insufficient response options (e.g. response options of ‘never’ and ‘no chance at all’ were difficult to distinguish) and insufficient instructions regarding how to answer certain items (Cassidy et al., 2020; Mason et al., 2022; Nicolaidis et al., 2020). Qualitative research by Mason et al. (2022) has suggested that these issues may, in part, be related to autistic adults’ literal interpretations of what are perceived to be ambiguous questions. For example, asking adults ‘are you able to accept your bodily appearance?’ was perceived to be problematic as autistic adults felt that physical appearance cannot be meaningfully changed.

To address these issues, previous studies have developed autism-specific items or adjusted wording and/or response options of existing items to create bespoke measures for autistic adults (Cassidy et al., 2021; McConachie et al., 2018; Nicolaidis et al., 2020; Rodgers et al., 2020). Sometimes, these changes included creative methods such as adding graphics to represent each response option and hotlinks to allow respondents to click and check the meaning/example of the terms (Nicolaidis et al., 2020). Given the views of participants in the present study, we suggest a similar process is undertaken for measures to address loneliness in autistic adults.

It is important to emphasise that participants raised issues with both measures that we evaluated in the current study. In addition, participants were not directly asked their preference for one scale over the other. That said, among our sample, there appeared to be a slight preference for the UCLA scale over the SELSA. Such responses noted how the UCLA scale was shorter and less frequently impacted by ambiguous wording. In the absence of direct quantitative data expressing community preferences on this matter, we cautiously suggest that researchers may want to consider the UCLA scale as a basis for adaptation for autistic adults.

Building on our own findings and the findings of previous studies that have addressed such issues in other existing measures, we suggest several ways in which the UCLA scale could be adapted. Specifically, we recommend the following to increase the specificity of the questions:

a. To disambiguate being autistic and being alone from being lonely (e.g., changing *‘How often do you feel alone?’* to *‘How often do you feel lonely?’*),
b. To indicate which relationships are being asked about (e.g., *‘In considering the questions, feel free to consider any kinds of relationships you may have in your life. They could be those that are close in proximity, online or with non-humans such as pets/animals.’*),
c. To clarify the parameters of the various response options (e.g., ‘*“never” means around 0% of the time (this may not apply to some people), “sometimes” means around 50% of the time, “always” means around 100% of the time’*)
d. To specify the time scale to consider (e.g., *‘please consider your experiences during the last month.’*) (see Umagami, 2023 for more detailed suggestions).

The proposed adaptations will need further discussion and development with the autistic community (as per Nicolaidis et al., 2020) but perhaps offer a starting point for such conversations. It is important to note, however, that the UCLA scale and the SELSA are fundamentally different measures of loneliness. While the UCLA scale is a unidimensional measure assessing loneliness as a global construct, the SELSA is a multidimensional measure assessing several dimensions of loneliness with three subscales: romantic subscale, family and social subscales (Cacioppo et al., 2015). The simplicity of the unidimensional measure appears to have been preferred by autistic adults, although the scores will therefore provide more limited insight into the experiences of respondents.

### Limitations

When considering the results of the present study, care should be taken regarding the generalisability of the findings. Despite directly contacting Black, Asian and Minority Ethnic (BAME) groups, participants in this study were predominantly of White ethnicity (90.1%, higher than the proportion of White individuals in the UK population, Office for National Statistics, 2021). These demographics do not reflect the UK autistic population as a whole, where the prevalence of autism was reported as highest for Black pupils (Roman-Urrestarazu et al., 2021). Furthermore, most participants (69%) had gained university qualifications despite autistic people facing significant barriers that often result in them ‘dropping out’ of university (Gurbuz et al., 2019; Newman et al., 2011). Given that the study involved completing an online measure and providing written responses to open questions, it is not surprising that most participants (93.1%) used spoken language to communicate, and thus this study may not represent the experiences of loneliness felt by autistic adults who use alternative communicative tools (e.g. sign language, communication devices). Nonetheless, the findings offer important insight for a group of participants who may be overlooked with respect to loneliness: autistic individuals without intellectual disability who can communicate verbally.

In addition, while the results of this study appeared to corroborate existing studies that suggested higher levels of loneliness in autistic adults compared to the general population, our study was without a comparison group, and therefore, results need to be treated with caution. As people tend to participate in survey studies on topics related to their interests (Groves et al., 2004), participants in the present study might therefore have been those who were more aware of their experiences of loneliness and/or were lonelier than the broader autistic population (and the general population more widely).

It should also be noted that while the UCLA scale and SELSA were chosen as the targets for our evaluation (based on how frequently these had been used with autistic adults in prior research), there are a number of alternative measures available. The findings of the present study could be used to inform a review of additional loneliness measures, focusing on aspects that were highlighted as problematic in the UCLA scale and SELSA. In this way, an optimal candidate for modification could be identified. As we have done here, it will also be important to ensure that the scores on such a measure map onto autistic people’s subjective experiences (e.g. on the direct measure of loneliness).

## Conclusion

Our findings suggest that while scores on the UCLA scale and SELSA may align with autistic adults’ subjective experiences of loneliness, autistic adults felt that both measures were difficult for them to complete. Specifically, there were concerns (1) that these loneliness measures do not reflect the nuance of autistic experience and instead are drawing conclusions based on neurotypical norms and (2) that the lack of clarity around questionnaire items may lead to interpretation of items in ways that the original measures did not intend. Further investigation – in collaboration with autistic adults – is needed to adapt loneliness measures to make them more acceptable, and appropriate, for the autistic population.

## Supplemental Material

sj-docx-1-aut-10.1177_13623613231217056 – Supplemental material for Evaluating measures to assess loneliness in autistic adults Supplemental material, sj-docx-1-aut-10.1177_13623613231217056 for Evaluating measures to assess loneliness in autistic adults by Kana Grace, Anna Remington, Jade Davies and Laura Crane in Autism

## Notes

1. In the present study, one of the response options in the UCLA scale (‘often’) was worded differently from the wording in the original paper (‘always’) (Russell, 1996). This was how the scale was presented in other existing resources for use in research (Fetzer Institute, n.d.). This change in response options from the original article might have impacted how autistic adults scored and commented on the scale. Indeed, some participants in this study confirmed that the response option ‘always’ would be appropriate to include. We suggest that future research should follow the scale presented in the original article and have the response option of ‘always’.
