# Supplementary material for: Evaluating measures to assess loneliness in autistic adults
Source: Autism. 2023 Dec 25;28(8):1959–71. doi: 10.1177/13623613231217056 (PMC11301961; doi:10.1177/13623613231217056)
Supplement: sj-docx-1-aut-10.1177_13623613231217056 – Supplemental material for Evaluating measures to assess loneliness in autistic adults [file sj-docx-1-aut-10.1177_13623613231217056.docx]

| **Supplementary Materials**  **Supplementary Material 1.** Full participant demographics (n= 203) | | |
| --- | --- | --- |
| Demographic variables |  | n (%) |
| Autism diagnosis | Formally diagnosed | 172 (84.7) |
|  | Self-identified and in process of obtaining a diagnosis | 14 (6.9) |
|  | Self-identified but not seeking a diagnosis | 17 (8.4) |
|  |  |  |
| Age of autism diagnosis (N=172) | M: years (SD) | 34.7 (14.8) |
|  | Range: years | 1-70 |
|  | Diagnosed in childhood (<18 years old) | 30 (17.4) |
|  | Diagnosed in adulthood | 142 (82.6) |
| Age | M: years (SD) | 40.7 (12.5) |
|  | Range: years | 18-73 |
|  | Aged 24 and younger | 28 (13.8) |
|  | Aged 25-34 | 35 (17.2) |
|  | Aged 35-44 | 58 (28.6) |
|  | Aged 45-54 | 50 (24.6) |
|  | Aged 55-65 | 28 (13.8) |
|  | Aged 66 and above | 4 (2.0) |
| Gender | Male (including transgender male) | 65 (32.0) |
|  | Female (including transgender female) | 116 (57.1) |
|  | Non-binary | 17 (8.4) |
|  | Other/prefer not to say | 5 (2.5) |
| Ethnicity | White (including British, Irish or any other White background) | 183 (90.1) |
|  | Black or Black British Caribbean (including the Caribbean, African or any other Black background) | 1 (0.5) |
|  | Asian or Asian British (including Indian, Pakistan, Bangladesh or any other Asian Background) | 4 (2.0) |
|  | Mixed (e.g., White and Asian; or any other Mixed background) | 10 (4.9) |
|  | Other/prefer not to say | 5 (2.5) |
| Location of growing up | In the UK | 180 (88.7) |
|  | In another country | 23 (11.3) |
| Co-occurring conditions | Yes | 144 (70.9) |
|  | Yes, but I prefer not to disclose the diagnosis/diagnoses | 9 (4.4) |
|  | No | 50 (24.6) |
| Co-occurring conditions (text-entry) *multiple answers were allowed (n=144) | Depression | 67 (33.0) |
|  | Anxiety | 58 (28.6) |
|  | Physical conditions | 51 (25.1) |
|  | Learning disabilities | 27 (13.3) |
|  | AD(H)D | 23 (11.3) |
|  | OCD | 10 (4.9) |
|  | Eating disorders | 8 (3.9) |
|  | PTSD | 7 (3.4) |
|  | Tourette’s syndrome | 2 (1.0) |
|  | Other mental health conditions (e.g., Bipolar disorder, Personality disorders) | 18 (8.9) |
| Ways of communication | Spoken language | 189 (93.1) |
|  | Sign language | 4 (2.0) |
|  | Communication devices and apps | 10 (4.9) |
| Living arrangement | Living with parents and/or siblings | 25 (12.3) |
|  | Living with non-family members (including living in university accommodation) | 19 (9.4) |
|  | Living with partner and/or children | 95 (46.8) |
|  | Living alone | 57 (28.1) |
|  | Living in a residential home | 1 (0.5) |
|  | Other (e.g., combination of the above) | 6 (3.0) |
| Employment | Full-time paid job (over 35 hours per week) | 65 (32.0) |
|  | Part-time paid job (less than | 33 (16.3) |
|  | Not employed – in full/part-time education (student) | 29 (14.3) |
|  | Not employed – not in education | 41 (20.2) |
|  | In voluntary employment | 15 (7.4) |
|  | Self-employed | 9 (4.4) |
|  | Retired | 2 (1.0) |
|  | Other (e.g., combination of the above) | 9 (4.4) |
| Educational history | Mainstream school | 189 (93.1) |
|  | Special unit within a mainstream school | 2 (1.0) |
|  | Specialist school | 6 (3.0) |
|  | Home school | 0 (0) |
|  | Other/prefer not to say | 6 (3.0) |
| Highest educational qualification^1^ | GCSEs | 11 (5.4) |
|  | National Vocational Qualification | 10 (4.9) |
|  | A/AS Levels | 26 (12.8) |
|  | First degree (e.g., BA, BSc) | 60 (29.6) |
|  | Higher degree (e.g., MA, MSc, PhD) | 80 (39.4) |
|  | Other/no qualifications | 16 (7.9) |
| Relationship | Married/in civil partnership | 53 (26.1) |
|  | Not married, but in a romantic relationship | 43 (21.2) |
|  | Divorced/separated | 19 (9.4) |
|  | Single | 87 (42.9) |
|  | Other (divorced and living with a new partner) | 1 (0.5) |
| Disclosure of autism diagnosis | Tend to share with anyone I meet | 41 (20.2) |
|  | Tend to share with those close to me (family, friends, co-workers, etc.) | 104 (51.2) |
|  | Tend not to share with anyone I meet including those close to me | 27 (13.3) |
|  | Tend to share if needed/relevant | 18 (8.9) |
|  | Other (e.g., when people are knowledgeable about autism) | 11 (5.4) |
|  | Do not tend to share | 2 (1.0) |

**Supplementary Material 2.** Full outputs for independent samples t-test and equivalent nonparametric tests

|  | UCLA | SELSA | Direct measure |
| --- | --- | --- | --- |
| Statistical test | Mann-Whitney test (non-parametric) | Independent samples t-test | Mann-Whitney test (non-parametric) |
| *p* | .24 | .94 | .90 |
| t-value or Mann-Whitney U | *U*=2311.00, *z*=-1.18 | t(201)=.07 | *U*=3126.00, *z*=-.13 |
| Formally diagnosed group | *Mdn*=61.00 | M=149.00, SE=44.61 | *Mdn*=2.00 |
| Self-identified group | *Mdn*=65.00 | M=148.39, SD=38.01 | *Mdn*=2.00 |

**Supplementary Material 3.** Table of themes and illustrative quotes

| **Themes** | **Sub-themes** | **Illustrative Quotes** |
| --- | --- | --- |
| 1. Conflating loneliness and autism | - 1. *Sociability norms* | “The questions [in the UCLA scale] appear to be completely ignoring the fact that I may be taking steps to actively avoid people” (P171, the UCLA scale)  “It needs to be adapted for autistic people. For example, Q11 How often do you feel left out? Well, that's never for me because I'm quite happy to be on my own, so I never feel left out. There's nothing for me to feel left out of because I'm not looking for friendship.” (P131, the UCLA scale)  “It [SELSA] assumes that aloneness is loneliness. They're not the same thing” (P101, SELSA)  “why are these question [in SELSA] always Neurotypical in nature” (P104, SELSA) |
|  | - 1. *Similarity to others* | “I also rarely feel 'outgoing and friendly' because I do not have a strong interest in socialising. I am not interested in most other human beings around me.” (P171, the UCLA scale)  “Being unable to engage, or refer to a shared experience or world view - because there isn't one. It's like being in a group where everyone speaks a common language - except you. It's like looking at the world through a shop window.” (P67, the UCLA)  “they [family members] don't understand me or have much in common with me” (P89, SELSA) |
|  | - 1. *Limited representation of relationships* | “At work (both my paid work and my voluntary work) I tend to feel supported by those around with and we have a common interest but they are not my friends.” (P39, the UCLA)  “I could have answered very differently depending on whether I was thinking about my mostly online relationships with friends and colleagues where I used to live compared with relationships in person with people where I live now” (P189, the UCLA)  “Also it [SELSA] doesn’t ask about pets which can help mitigate loneliness so doesn’t give the whole picture” (P22, SELSA) |
|  | - 1. *The impact of camouflaging* | “and the effort that it takes to come across as 'outgoing'” (P171, the UCLA scale)  “the hassle to have fake smalltalks and all the awkward social instances” (P4)  “I have found 'friendships' to be largely exhausting and always requiring compromise such as masking on my part.” (P91, SELSA) |
| 1. Loneliness is not a static trait | - 1. *Contextual aspect of loneliness* | “It [the UCLA scale] could ask questions about context. E.g. I don't feel lonely when I'm with partner but feel very lonely when in a group at work” (P9, the UCLA scale)  “I also think there is a difference between situations, for example at home, at work, in social situations - these are all different and the loneliness I feel varies sometimes depending on where I am” (P103, the UCLA scale)  In relation to SELSA, “I wanted to say it depends or what does that mean or in some circumstances this and others that ... how can I answer a yes or no question when it varies” (P158, SELSA) |
|  | - 1. *Loneliness will change over time* | “My experience of loneliness is very different now to when I was a child. Now I am happily married although I have few friends other than my wife. In my childhood I mostly had no friends at all” (P18, the UCLA scale)  “Also [the UCLA scale] doesn’t deal with the history of lonely feeling” (P291, the UCLA scale)  “All of these tests read like loneliness is a constant feeling you have that doesn't vary over time, situation or context” (P140, SELSA) |
| 1. Unclear wording | - 1. *Lack of clarity* | “I am being asked to judge all people as the same. I feel in tune with other autistic activists. I am not in tune with the general population.” (P85, the UCLA scale)  “Some expressions will not be understood by some ('in tune', 'companionship')” (P80, the UCLA scale)  “would appreciate it if the terms used were defined more clearly, for example should I be including my girlfriend as a friend?” (P183, SELSA) |
|  | - 1. *The response options were inadequate* | “Perhaps you could create a modified version for the autistic population which includes the option, 'I don't do this', for all questions?” (P66, the UCLA scale)  “Having an option that says I don't know” (P93, SELSA)  “I had to answer a few questions As If I didn't have a close friend... I sort of wished for a 'Not Applicable' option for the questions!” (P137, SELSA) |
|  | - 1. *Compound questions were difficult for respondents to answer accurately* | “Some questions need you to agree/disagree with different parts of them to answer, which is confusing” (P68, SELSA)  “also the questions in effect cover two aspects eg Q8 is about whether we have partners (say) and then how positive our feelings are for those partners” (P129, SELSA)  “The questions repeatedly combine more than one factor yet only allow for a singe response e.g. Q4 I have a romantic partner with whom I share my most intimate thoughts and feelings. I don't know how to respond to this. I have a romantic partner with whom I share some thoughts and feelings that I would not share with anyone else. Are these my most intimate thoughts and feelings? Probably not.” (P186, SELSA) |
